# Incidence and prevalence of hypereosinophilic syndrome in the United States: A retrospective claims database study^☆^

**DOI:** 10.1016/j.waojou.2026.101451

**Published:** 2026-09-09

**Authors:** Bo Ding, Thanai Pongdee, Paul Dolin, Priya Jain, Jessica F. Most, Dylan Taylor, Ruth Owen, Lyndsi Licona, Itzíar Ubillos, Ignacio Méndez, Stephanie Yanjing Chen

**Affiliations:** a BioPharmaceuticals Medical, AstraZeneca, Gothenburg, Sweden; b Division of Allergic Diseases, Mayo Clinic, Rochester, MN, USA; c BioPharmaceuticals Medical, AstraZeneca, Cambridge, UK; d BioPharmaceuticals Medical, AstraZeneca, Wilmington, DE, USA; e Oxon Epidemiology, London, UK; f Oxon Epidemiology, Madrid, Spain; g BioPharmaceuticals Medical, AstraZeneca, Gaithersburg, MD, USA

**Keywords:** Hypereosinophilic syndrome, Eosinophils, Incidence, Prevalence, Health insurance

## Abstract

**Background:**

Hypereosinophilic syndrome (HES) encompasses rare disorders characterized by persistent hypereosinophilia resulting in organ damage. This study assessed demographics, incidence, and prevalence of HES in the United States following the introduction of HES-specific International Classification of Diseases 10th revision, Clinical Modification (ICD-10-CM) codes in 2020.

**Methods:**

This retrospective analysis used data from patients with HES and ≥12 months of continuous insurance coverage between October 1, 2020, and June 30, 2024, from Optum's de-identified Clinformatics® Data Mart Database. Incidence rates were calculated from the number of new HES cases divided by total person-time at risk, and prevalence from the proportion of HES cases in the population during the study.

**Results:**

Overall, 695 and 1224 patients fulfilled the incident and prevalent HES case definition, respectively. The overall HES incidence rate (95% CI) was 2.01 (1.87–2.17) per 100,000 person-years, with fluctuations between 2021 and 2024. The overall HES prevalence (95% CI) was 5.32 (5.02–5.62) per 100,000 persons, with increases between 2020 and 2024. Incidence and prevalence increased with age, with an almost even female-to-male ratio (51:49).

**Conclusions:**

HES had low incidence and prevalence, with increasing prevalence from 2020 to 2024, indicating an increased recording and growing awareness of HES with ICD-10-CM codes usage.

## Introduction

Hypereosinophilic syndrome (HES) encompasses a rare and complex group of heterogeneous disorders characterized by a persistent absolute eosinophil count of ≥1500 cells/μL leading to eosinophil-mediated organ damage.^1^, ^2^, ^3^ HES subtypes are classified by clinical features and underlying etiology.^1^ Few data are available on the incidence and prevalence of HES, largely due to the lack of specific International Classification of Diseases (ICD) codes. In the United States, HES-specific codes were introduced in the ICD 10th Revision, Clinical Modification (ICD-10-CM) in October 2020, enabling a more accurate estimation of HES incidence and prevalence. This study is the first to estimate HES incidence and prevalence using the new HES ICD-10-CM codes.

## Methods

### Study design

This was a retrospective analysis using data from the Optum's de-identified Clinformatics® Data Mart Database of commercial and Medicare Advantage supplemental health insurance plans and administrative claims between October 1, 2020, and June 30, 2024.

### Study population

The study population comprised all patients recorded in the Optum Clinformatics® with known sex, year of birth, and ≥12 months of continuous insurance coverage between October 1, 2020, to June 30, 2024. The study started from the addition of unique ICD-10-CM codes for HES (D72.11) and its subtypes (D72.110 for idiopathic HES [I-HES], D72.111 for lymphocytic HES [L-HES], D72.118 for other HES, and D72.119 for HES type unspecified) in the United States. End of follow-up coincided with the June 30, 2024 database update. A HES case was defined as a patient having ≥1 inpatient or ≥2 outpatient diagnoses of HES ≥4 weeks apart from the beginning of the study. An incident HES case was any patient who met the above HES definition, with no prior HES records before the index date (defined as the latest date from October 1, 2021 to the date the patient fulfilled the inclusion criteria). A prevalent HES case was any patient who met the above HES definition, with a HES diagnosis anytime from October 1, 2020, to June 30, 2024.

### Statistical analyses

Overall annual prevalence and incidence rates were calculated. Sensitivity analyses on a less restrictive definition of HES case and shorter insurance coverages were performed. Detailed information on statistical analyses are described in the Supplemental Methods.

## Results

### Incidence

A total of 21,990,978 patients fulfilled the inclusion criteria for the incidence population, with 695 patients meeting the incident HES case definition (Table 1). The incident HES cohort had a mean (standard deviation [SD]) age of 65.9 (17.5) years and 51.1% were female. Of the incident HES cases with race recorded, patients were predominantly White (62.0%). The distribution of HES subtypes were I-HES (28.3%), L-HES (2.9%), other HES (17.6%), and unspecified HES (51.2%).

**Table 1:** Patient demographics of incident and prevalent populations, and HES cohorts overall and by HES subtype.

Incident patients						
	Overall incident population	Overall incident HES cohort	I-HES	L-HES	Other HES	Unspecified HES
	(N = 21,990,978)	(N = 695)	(n = 197)	(n = 20)	(n = 122)	(n = 356)
Subtype distribution	NA	100%	28.3%	2.9%	17.6%	51.2%
**Age (continuous), years**^a^						
Mean (SD)	50.3 (23.9)	65.9 (17.5)	66.1 (17.6)	69.2 (16.8)	68.9 (14.4)	64.6 (18.4)
Median	54.0	70.0	71.0	74.0	71.0	70.0
(Q1, Q3)	(31.0, 70.0)	(60.0, 77.0)	(61.0, 77.0)	(61.5, 80.0)	(66.0, 77.0)	(56.5, 77.0)
**Age (categorical), years**^a^						
<20	3,022,875 (13.7%)	21 (3.0%)	7 (3.6%)	0 (0%)	2 (1.6%)	12 (3.4%)
20–29	2,060,894 (9.4%)	12 (1.7%)	3 (1.5%)	1 (5.0%)	1 (0.8%)	7 (2.0%)
30–39	2,498,229 (11.4%)	26 (3.7%)	5 (2.5%)	1 (5.0%)	1 (0.8%)	19 (5.3%)
40–49	2,296,520 (10.4%)	48 (6.9%)	15 (7.6%)	1 (5.0%)	9 (7.4%)	23 (6.5%)
50–59	2,425,476 (11.0%)	65 (9.4%)	18 (9.1%)	0 (0%)	5 (4.1%)	42 (11.8%)
60–69	3,918,493 (17.8%)	153 (22.0%)	46 (23.4%)	4 (20.0%)	32 (26.2%)	71 (19.9%)
70–79	3,842,684 (17.5%)	243 (35.0%)	67 (34.0%)	8 (40.0%)	50 (41.0%)	118 (33.1%)
≥80	1,925,807 (8.8%)	127 (18.3%)	36 (18.3%)	5 (25.0%)	22 (18.0%)	64 (18.0%)
**Sex**						
Female	11,433,630 (52.0%)	355 (51.1%)	100 (50.8%)	6 (30.0%)	58 (47.5%)	191 (53.7%)
Male	10,557,348 (48.0%)	340 (48.9%)	97 (49.2%)	14 (70.0%)	64 (52.5%)	165 (46.3%)
**Race**						
African American	1,660,828 (7.6%)	55 (7.9%)	12 (6.1%)	<5	7 (5.7%)	33 (9.3%)
Asian	658,387 (3.0%)	19 (2.7%)	<5	0 (0%)	7 (5.7%)	11 (3.1%)
White	10,705,801 (48.7%)	431 (62.0%)	125 (63.5%)	14 (70.0%)	75 (61.5%)	217 (61.0%)
Unknown	8,965,962 (40.8%)	190 (27.3%)	59 (29.9%)	3 (15.0%)	33 (27.0%)	95 (26.7%)
**Region**^b^						
South	9,088,619 (41.3%)	253 (36.4%)	76 (38.6%)	12 (60.0%)	41 (33.6%)	124 (34.8%)
Midwest	5,362,055 (24.4%)	152 (21.9%)	41 (20.8%)	<5	23 (18.9%)	84 (23.6%)
West	4,767,705 (21.7%)	164 (23.6%)	45 (22.8%)	<5	27 (22.1%)	91 (25.6%)
Northeast	2,433,443 (11.1%)	124 (17.8%)	35 (17.8%)	<5	30 (24.6%)	56 (15.7%)
Unknown	339,156 (1.5%)	2 (0.3%)	0 (0%)	0 (0%)	1 (0.8%)	1 (0.3%)

**Prevalent patients**						
	Overall prevalent population	Overall prevalent HES cohort	I-HES	L-HES	Other HES	Unspecified HES
	(N = 23,015,204)	(N = 1224)	(n = 367)	(n = 32)	(n = 208)	(n = 617)
Subtype distribution	NA	100%	30.0%	2.6%	17.0%	50.4%
**Age (continuous), years**^a^						
Mean (SD)	49.0 (23.8)	63.6 (18.1)	64.0 (17.6)	61.6 (21.9)	66.6 (15.9)	62.5 (18.8)
Median (Q1, Q3)	53.0 (30.0, 69.0)	68.0 (56.0, 76.0)	68.0 (55.0, 76.0)	70.0 (49.5, 77.0)	70.0 (62.0, 76.5)	68.0 (53.0, 76.0)
**Age (categorical), years**^a^						
<20	3,359,584 (14.6%)	42 (3.4%)	11 (3.0%)	3 (9.4%)	5 (2.4%)	23 (3.7%)
20–29	2,288,233 (9.9%)	38 (3.1%)	12 (3.3%)	1 (3.1%)	4 (1.9%)	21 (3.4%)
30–39	2,647,061 (11.5%)	56 (4.6%)	13 (3.5%)	2 (6.3%)	3 (1.4%)	38 (6.2%)
40–49	2,418,513 (10.5%)	89 (7.3%)	27 (7.4%)	2 (6.3%)	15 (7.2%)	45 (7.3%)
50–59	2,588,561 (11.2%)	142 (11.6%)	45 (12.3%)	3 (9.4%)	18 (8.7%)	76 (12.3%)
60–69	4,287,438 (18.6%)	293 (23.9%)	89 (24.3%)	4 (12.5%)	55 (26.4%)	145 (23.5%)
70–79	3,704,173 (16.1%)	378 (30.9%)	114 (31.1%)	11 (34.4%)	73 (35.1%)	180 (29.2%)
≥80	1,721,641 (7.5%)	186 (15.2%)	56 (15.3%)	6 (18.8%)	35 (16.8%)	89 (14.4%)
**Sex**						
Female	11,954,173 (51.9%)	624 (51.0%)	189 (51.5%)	11 (34.4%)	99 (47.6%)	325 (52.7%)
Male	11,061,031 (48.1%)	600 (49.0%)	178 (48.5%)	21 (65.6%)	109 (52.4%)	292 (47.3%)
**Race**						
African American	1,740,264 (7.6%)	115 (9.4%)	32 (8.7%)	<5	19 (9.1%)	61 (9.9%)
Asian	685,954 (3.0%)	35 (2.9%)	7 (1.9%)	0 (0%)	10 (4.8%)	18 (2.9%)
White	11,130,929 (48.4%)	753 (61.5%)	224 (61.0%)	24 (75.0%)	129 (62.0%)	376 (60.9%)
Unknown	9,458,057 (41.1%)	321 (26.2%)	104 (28.3%)	5 (15.6%)	50 (24.0%)	162 (26.3%)
**Region**^b^						
South	9,556,939 (41.5%)	466 (38.1%)	134 (36.5%)	17 (53.1%)	77 (37.0%)	238 (38.6%)
Midwest	5,620,659 (24.4%)	276 (22.5%)	77 (21.0%)	7 (21.9%)	43 (20.7%)	149 (24.1%)
West	4,950,272 (21.5%)	273 (22.3%)	89 (24.3%)	<5	44 (21.2%)	136 (22.0%)
Northeast	2,542,483 (11.0%)	206 (16.8%)	67 (18.3%)	<5	42 (20.2%)	93 (15.1%)
Unknown	344,851 (1.5%)	3 (0.2%)	0 (0%)	0 (0%)	2 (1.0%)	1 (0.2%)

HES, hypereosinophilic syndrome; I-HES, idiopathic hypereosinophilic syndrome; L-HES, lymphocytic hypereosinophilic syndrome; NA, not applicable; N/n, number of patients; Q1, first quartile; Q3, third quartile; SD, standard deviation.

a As of the index date.

b As of any point during the study period.

**Fig. 1:**
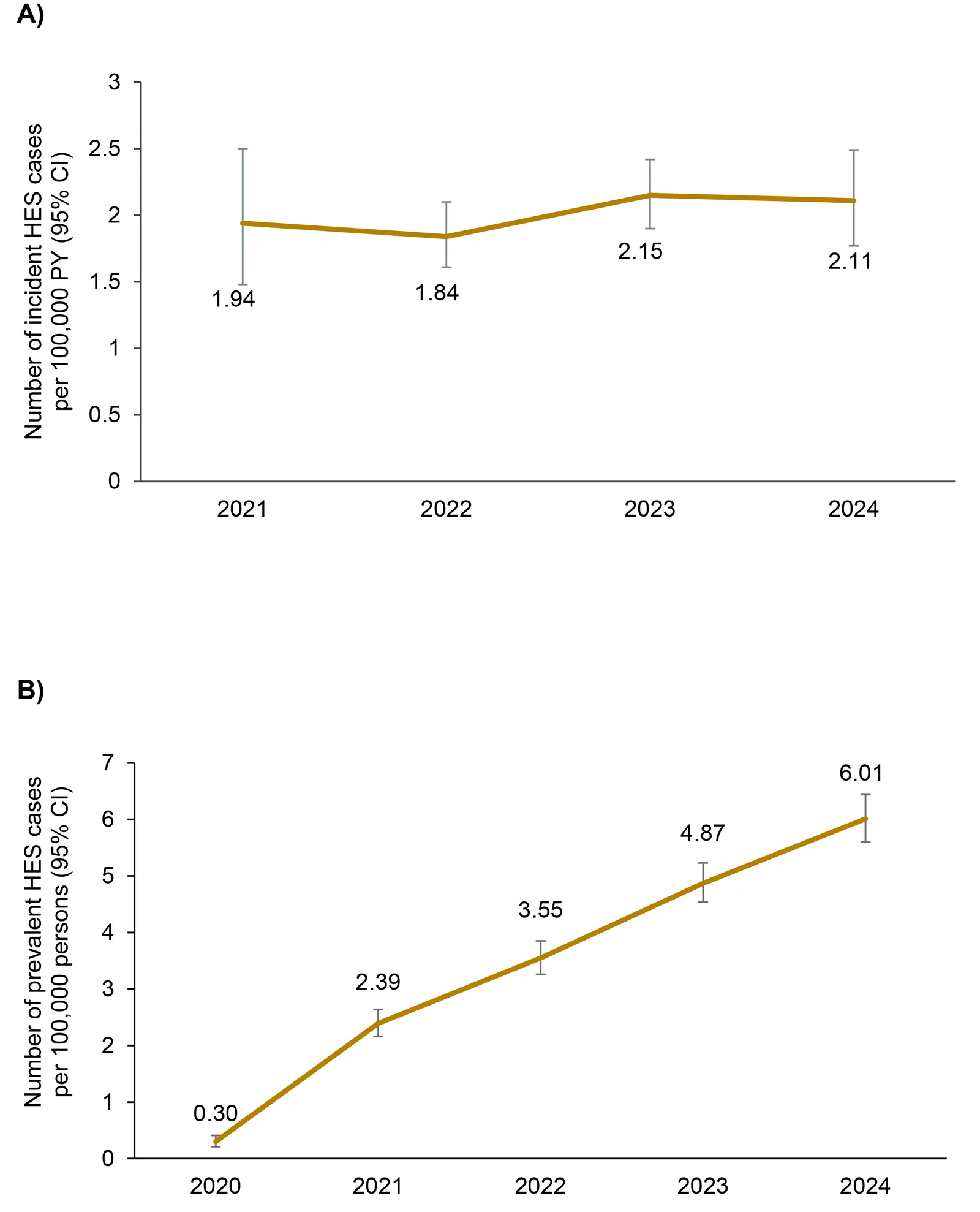
Annual overall HES incidence (A) and prevalence (B). CI, confidence interval; HES, hypereosinophilic syndrome; PY, person-years

The overall HES incidence rate (95% confidence interval [CI]) for the study period was 2.01 (1.87–2.17) per 100,000 person-years (PY) and was broadly similar in males (2.08 [1.87–2.32]) and females (1.95 [1.75–2.16]). The overall HES incidence (95% CI) for the study period increased with age: from 0.49 (0.30–0.76) per 100,000 PY in the <20 years age group to 3.49 (2.95–4.10) per 100,000 PY in the ≥80 years age group. The annual HES incidence (95% CI) fluctuated each year between 2021 and 2024, from 1.94 (1.48–2.50) per 100,000 PY in 2021 to 2.11 (1.77–2.49) per 100,000 PY in 2024 (Fig. 1A).

### Prevalence

A total of 23,015,204 patients fulfilled the inclusion criteria for the prevalence population, with 1224 patients meeting the prevalent HES case definition (Table 1). The prevalent HES cohort had a mean (SD) age of 63.6 (18.1) years and 51.0% were female. As with incident HES cases, the majority of patients who met the prevalent HES case definition were predominantly White (61.5%). The distribution of HES subtypes were I-HES (30.0%), L-HES (2.6%), other HES (17.0%), and unspecified HES (50.4%).

The overall HES prevalence (95% CI) for the study period was 5.32 (5.02–5.62) per 100,000 persons and was similar in males (5.42 [5.00–5.88]) and females (5.22 [4.82–5.65]). The overall HES prevalence (95% CI) for the study period increased with age: from 1.25 (0.90–1.69) per 100,000 persons in the <20 years age group to 10.80 (9.31–12.47) per 100,000 persons in the ≥80 years age group. The annual HES prevalence (95% CI) increased each year between 2020 and 2024, rising from 0.30 (0.21–0.41) per 100,000 persons in 2020 to 6.01 (5.60–6.44) per 100,000 persons in 2024 (Fig. 1B).

### Sensitivity analysis

A sensitivity analysis was performed using a single outpatient diagnosis and without ≥4 weeks follow-up history for annual overall HES incidence and prevalence (Fig. S1). An additional 40 incident HES cases were identified with slight variations from the original analyses noted for the earliest and latest years (Fig. S1A). Trends for an additional 45 prevalent HES cases were similar to the original annual overall HES prevalence analysis (Fig. S1B). Another sensitivity analysis accounting for 6 months of continuous insurance coverage was also performed. Analysis of incidence was not feasible as additional incident HES cases were not identified. However, 67 additional prevalent HES cases were identified, with slight decreases in annual overall HES prevalence noted after 2021 compared to the original analysis (Fig. S2).

## Discussion

To our knowledge, this is the first study to report the incidence and prevalence of HES annually, and overall by sex and age, in the United States using the newly introduced HES ICD-10-CM diagnostic codes from Optum Clinformatics®. We observed that overall HES incidence rates were higher than those previously noted in literature for the United States (0.18–0.36 per 100,000 PY).^4^ These differences may result from an underestimation of HES prevalence in earlier studies lacking HES-specific ICD-10-CM codes or from variations in data sources and time periods.^5^ Patients whose HES progressed may also be underrepresented due to mortality, insurance loss, or a shorter insurance coverage period.

Additionally, the slight fluctuations observed in annual overall HES incidence rates between 2021 and 2022 and increases in 2023 may have resulted from US physicians being in the process of adopting the new HES ICD-10-CM codes. This gradual increase in HES ICD-10-CM code usage (which appears to be ongoing), together with improved recognition and diagnostic accuracy, likely reflects greater disease awareness and may have contributed to the observed rise in prevalence over time. We found that the general trends in our findings align with previous studies from Europe where incidence fluctuated annually, while prevalence showed smaller and more gradual increases.^6^^,^^7^

## Conclusion

The data suggests that HES has a low overall incidence and prevalence. From 2020 to 2024, annual prevalence increased while incidence remained broadly stable, a pattern compatible with an accumulation of diagnosed chronic cases over time and an incremental uptake of HES-specific ICD-10-CM codes.

## Ethics statement

This study used data from Optum's de-identified Clinformatics® Data Mart Database. The data are compliant with the Health Insurance Portability and Accountability Act; therefore, Institutional Review Board approval was not required.

## Author contributions

Study conception and design: BD, PD, PJ, DT, RO, LL, IU, IM, and SYC; study conduct and data analysis: DT, RO, LL, IU, IM. All authors contributed to data interpretation, as well as the drafting and review of the publication. All authors gave their consent for the publication of this article.

## Data availability statement

The data that support the findings of this study are available from Optum, but restrictions apply to the availability of these data, which were used under license for the current study, and so are not publicly available.

## Submission declaration

The authors formally declare that the content of this paper is an original work. The authors note that this work has also been previously presented at the American Academy of Allergy, Asthma and Immunology Annual Meeting 2026. It is not under review by any other journal.

## Previous presentation

Presented at the following conference: Pongdee T et al. Incidence and prevalence of hypereosinophilic syndrome in the United States: a retrospective claims database study. American Academy of Allergy, Asthma & Immunology Annual Meeting 2026. Abstract Number: 245. Available at: https://www.jacionline.org/article/S0091-6749(25)01915-3/fulltext.

## Disclosure of the use of Generative AI and AI-assisted Technologies

Nothing to disclose.

## Funding

This study was funded by 10.13039/100004325AstraZeneca.

Role of the funding source: 10.13039/100004325AstraZeneca funded this study and participated in the study design, data interpretation, and writing of the study report. AstraZeneca reviewed the manuscript, without influencing the opinions of the authors, to ensure medical and scientific accuracy, and protection of intellectual property. All authors had the final responsibility for the decision to submit the manuscript for publication. AstraZeneca funded medical writing support for the development of this manuscript.

## Declaration of competing interest

The authors declare the following financial interests/relationships which may be considered as a potential conflict of interest: TP has received research funding from AstraZeneca, Blueprint Medicines, Cogent Biosciences, GlaxoSmithKline, Regeneron, and Sanofi. BD, PD, PJ, JFM, and SYC are or were employees or contractors of AstraZeneca at the time the retrospective analysis was performed and may hold stock in AstraZeneca. DT, RO, LL, IU, and IM are employees of Oxon Epidemiology, which received research funding from AstraZeneca for the conduct of this study.
