# Supplementary material for: Incidence and prevalence of hypereosinophilic syndrome in the United States: A retrospective claims database study
Source: World Allergy Organ J. 2026 Sep 9;19(9):101451. doi: 10.1016/j.waojou.2026.101451 (PMC13572994; doi:10.1016/j.waojou.2026.101451)
Supplement: Supplementary file 1 [file mmc1.pdf]

Supplemental appendix

**Title:** Incidence and prevalence of hypereosinophilic syndrome in the United States: a retrospective claims database study
**Short title:** Incidence and prevalence of hypereosinophilic syndrome in the US
**Authors:** Bo DING, PhD, Thanai PONGDEE, MD, Paul DOLIN, PhD, Priya JAIN, MD, Jessica F. MOST, MD, Dylan TAYLOR, MSc, Ruth OWEN, MSc, Lyndsi LICONA, MSc, Itzíar UBILLOS, PhD, Ignacio MÉNDEZ, MSc, Stephanie Yanjing CHEN, PhD
• Supplemental methods<br>o Statistical analyses……………………………………………..pg. 2<br>• Supplemental figures<br>o Fig. S1. Annual overall HES incidence (A) and prevalence (B) in the sensitivity vs original analysis for outpatient diagnoses.…...pg. 4<br>o Fig. S2. Annual overall HES prevalence in the sensitivity vs. original analysis for 6-month enrollment ……………….…………….pg. 5

Supplemental methods

Statistical analyses

Descriptive statistics were used to describe the demographic features and distribution of incident and prevalent HES cases. Covariates for age, gender, ethnicity/race, region, and year of diagnosis were used to assess demographic features of patients with hypereosinophilic syndrome (HES). Incidence rate and 95% confidence intervals (CIs) were estimated as the number of incident HES cases divided by the total person-time at risk during the study period and reported as the number of new cases per 100,000 person-years (PY) overall and annually, by gender, and by 10-year age groups. CIs were calculated using the Poisson exact method for incident cases. Prevalence and 95% CIs were estimated as the proportion of patients with HES in the study population and were reported as the number of cases per 100,000 persons overall and annually, by gender, and by 10-year age groups. CIs were calculated using the Clopper-Pearson method for prevalent cases.

Sensitivity analyses were also conducted to increase confidence in the overall methodology. To account for instances where a patient only had one outpatient diagnosis of HES and did not have ≥4 weeks of follow-up post-diagnosis to ascertain a qualifying second outpatient diagnosis of HES at the end of the study period (i.e. end of data capture), a sensitivity analysis was performed in which all qualifying HES cases and those without sufficient follow-up subsequent to an initial outpatient diagnosis were considered to have had HES. An additional sensitivity analysis was conducted for patients with a shortened continuous insurance coverage criterion of 6 months to assess if shortening the length of membership in the inclusion criteria

would result in the identification of additional patients. All programming was performed in SAS Version 9.4 (TS1M6).

Supplemental figures

Figure S1. Annual overall HES incidence (A) and prevalence (B) in the sensitivity vs original analysis for outpatient diagnoses

A)

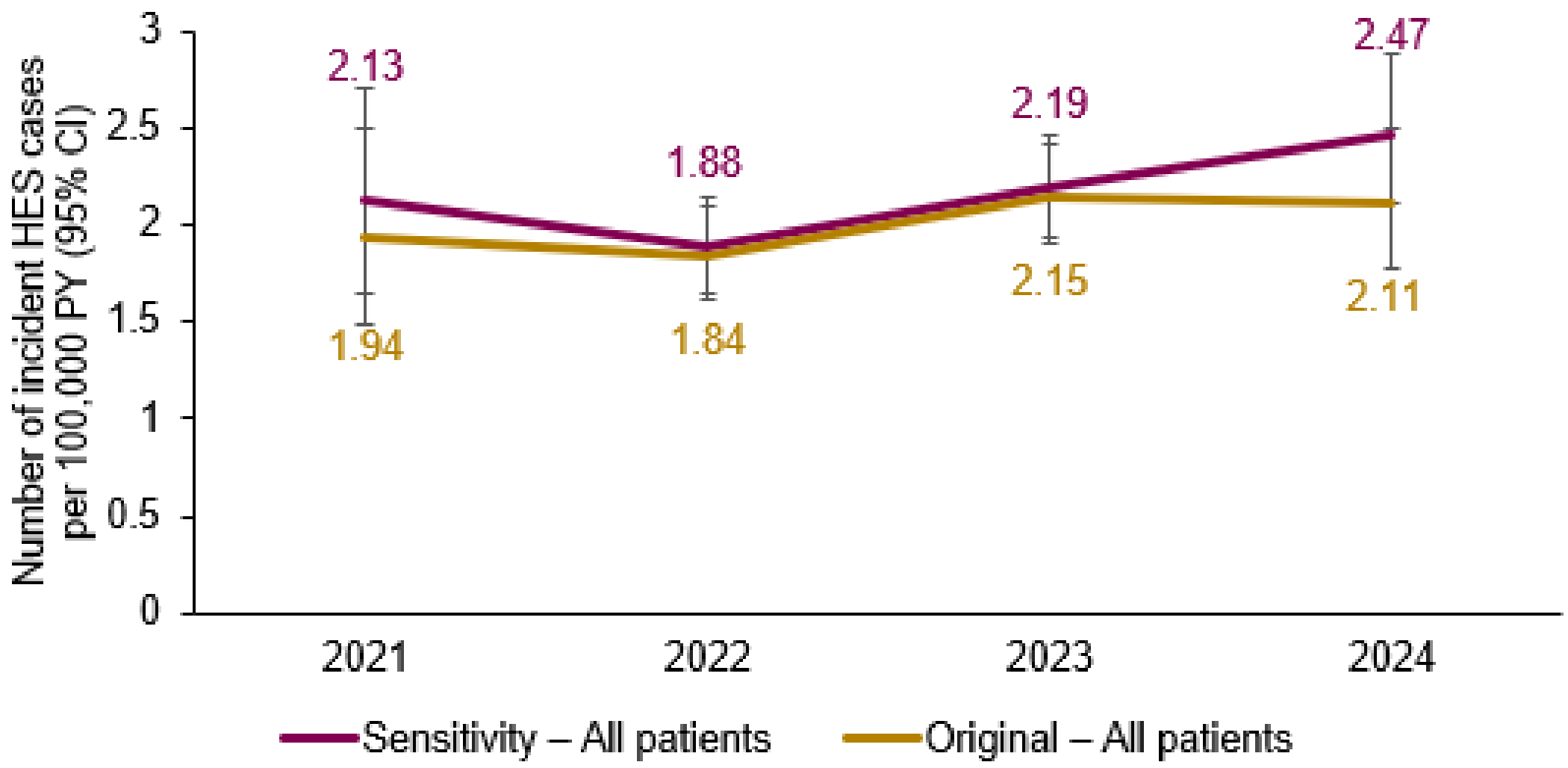

B)

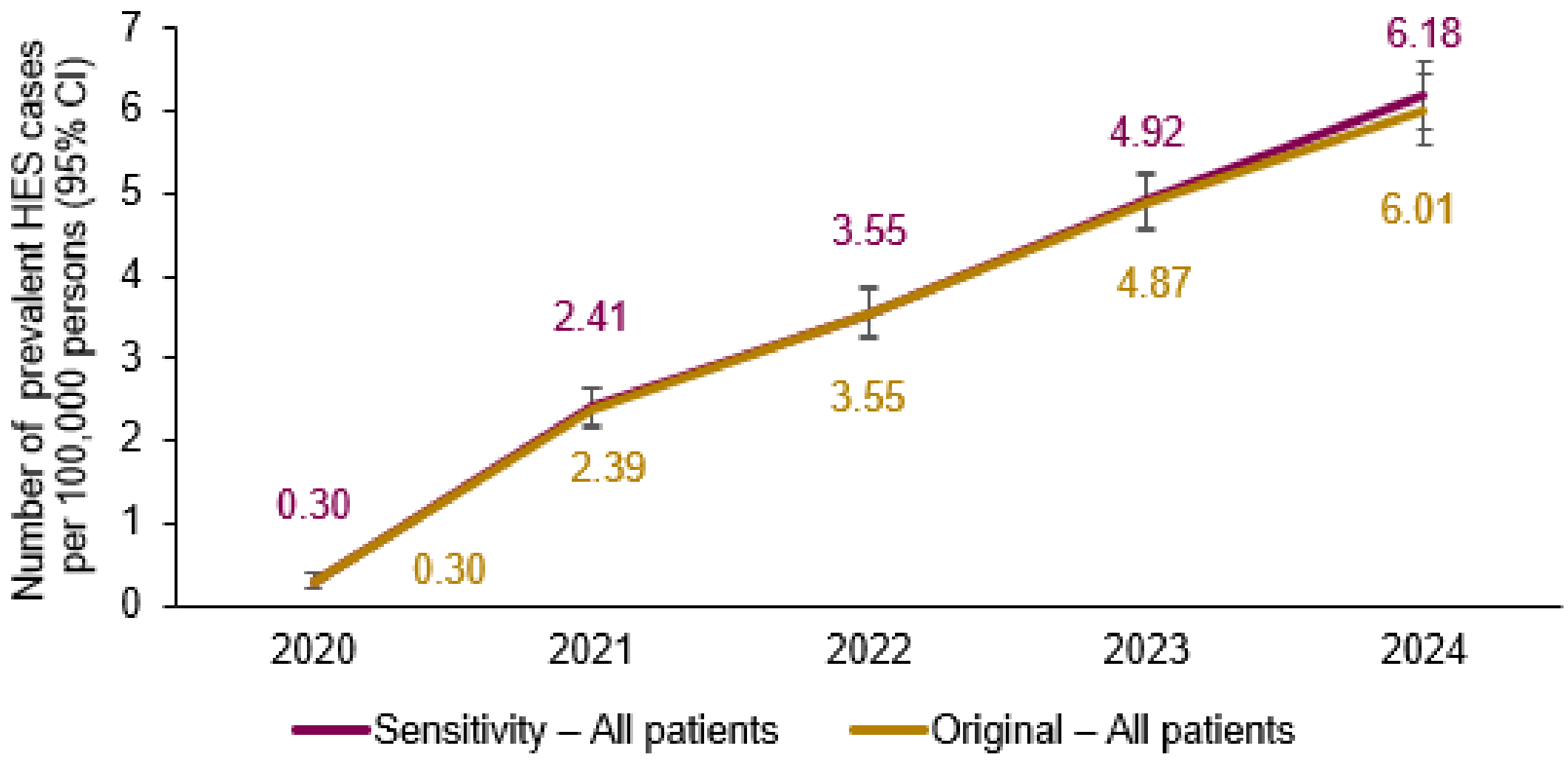

CI, confidence interval; HES, hypereosinophilic syndrome; PY, person-years.

Figure S2. Annual overall HES prevalence in the sensitivity vs. original analysis for 6-month enrollment

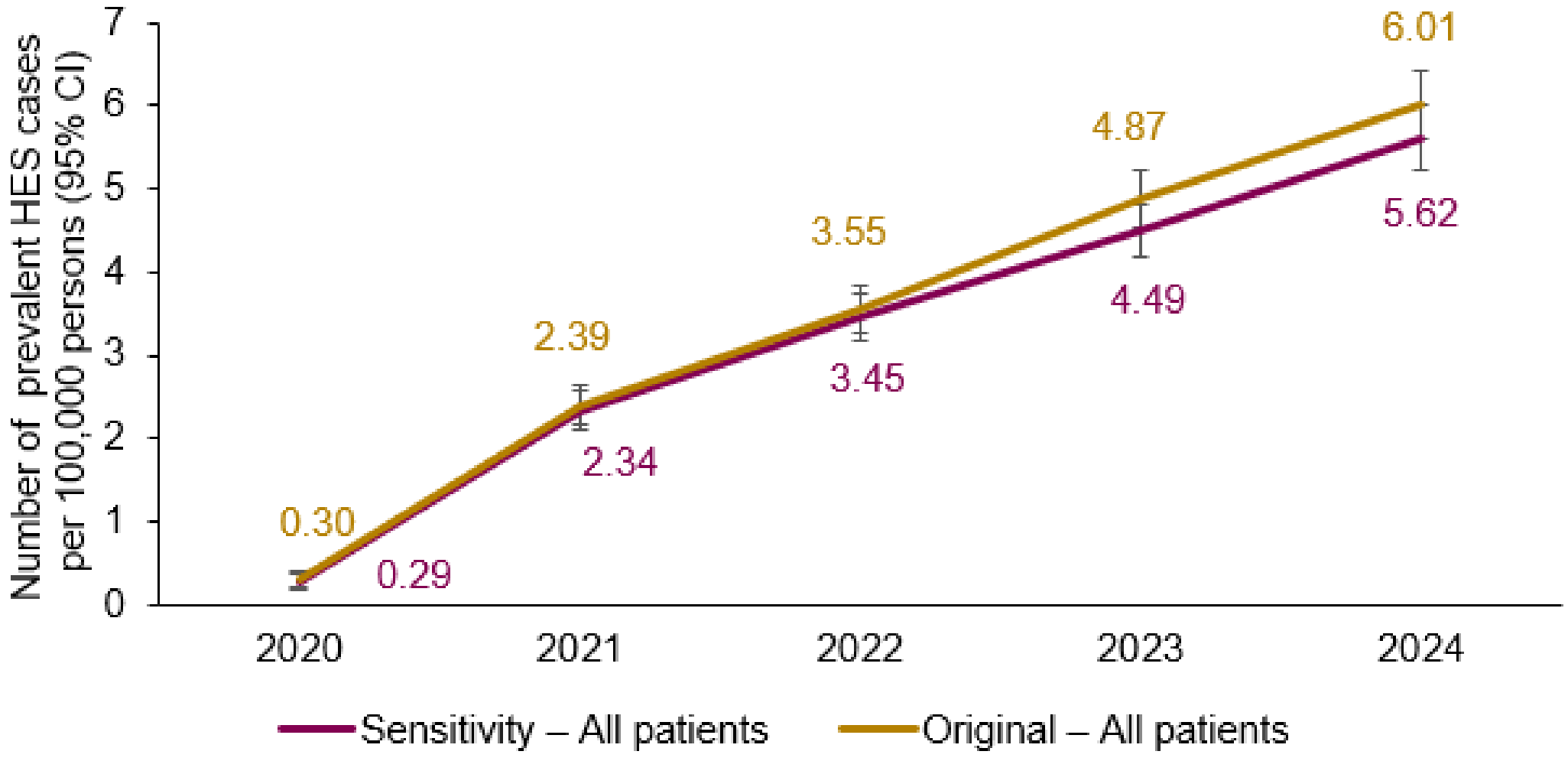

CI, confidence interval; HES, hypereosinophilic syndrome.
